# Hypoxia increases membrane metallo-endopeptidase expression in a novel lung cancer *ex vivo* model – role of tumor stroma cells

**DOI:** 10.1186/1471-2407-14-40

**Published:** 2014-01-25

**Authors:** Katharina Leithner, Christoph Wohlkoenig, Elvira Stacher, Jörg Lindenmann, Nicole A Hofmann, Birgit Gallé, Christian Guelly, Franz Quehenberger, Philipp Stiegler, Freyja-Maria Smolle-Jüttner, Sjaak Philipsen, Helmut H Popper, Andelko Hrzenjak, Andrea Olschewski, Horst Olschewski

**Affiliations:** 1 Division of Pulmonology, Department of Internal Medicine, Medical University of Graz, Auenbruggerplatz 20, A-8036 Graz, Austria; 2 Institute of Pathology, Medical University of Graz, Auenbruggerplatz 25, A-8036 Graz, Austria; 3 Division of Thoracic and Hyperbaric Surgery, Department of Surgery, Medical University of Graz, Auenbruggerplatz 29, A-8036 Graz, Austria; 4 Stem Cell Research Unit, Medical University of Graz, Stiftingtalstraße 24, A-8010 Graz, Austria; 5 Institute for Molecular Biology and Biochemistry, Medical University of Graz, Harrachgasse 21/III, A-8010 Graz, Austria; 6 Core Facility Molecular Biology, Center for Medical Research, Medical University of Graz, Stiftingtalstraße 24, A-8010 Graz, Austria; 7 Institute for Medical Informatics, Statistics and Documentation, Medical University of Graz, Auenbruggerplatz 2, A-8036 Graz, Austria; 8 Division of Transplant Surgery, Department of Surgery, Medical University of Graz, Auenbruggerplatz 29, A-8036 Graz, Austria; 9 Department of Cell Biology, Erasmus University Medical Center, PO Box 2040, 3000 CA Rotterdam, The Netherlands; 10 Experimental Anesthesiology, University Clinic for Anesthesiology and Intensive Care Medicine, Medical University of Graz, Auenbruggerplatz 29, A-8036 Graz, Austria; 11 Ludwig Boltzmann Institute for Lung Vascular Research, Stiftingtalstraße 24, A-8010 Graz, Austria

**Keywords:** Hypoxia, Tumor, Expression array, Prognosis

## Abstract

**Background:**

Hypoxia-induced genes are potential targets in cancer therapy. Responses to hypoxia have been extensively studied *in vitro*, however, they may differ *in vivo* due to the specific tumor microenvironment. In this study gene expression profiles were obtained from fresh human lung cancer tissue fragments cultured *ex vivo* under different oxygen concentrations in order to study responses to hypoxia in a model that mimics human lung cancer *in vivo*.

**Methods:**

Non-small cell lung cancer (NSCLC) fragments from altogether 70 patients were maintained *ex vivo* in normoxia or hypoxia in short-term culture. Viability, apoptosis rates and tissue hypoxia were assessed. Gene expression profiles were studied using Affymetrix GeneChip 1.0 ST microarrays.

**Results:**

Apoptosis rates were comparable in normoxia and hypoxia despite different oxygenation levels, suggesting adaptation of tumor cells to hypoxia. Gene expression profiles in hypoxic compared to normoxic fragments largely overlapped with published hypoxia-signatures. While most of these genes were up-regulated by hypoxia also in NSCLC cell lines, membrane metallo-endopeptidase (MME, neprilysin, CD10) expression was not increased in hypoxia in NSCLC cell lines, but in carcinoma-associated fibroblasts isolated from non-small cell lung cancers. High MME expression was significantly associated with poor overall survival in 342 NSCLC patients in a meta-analysis of published microarray datasets.

**Conclusions:**

The novel *ex vivo* model allowed for the first time to analyze hypoxia-regulated gene expression in preserved human lung cancer tissue. Gene expression profiles in human hypoxic lung cancer tissue overlapped with hypoxia-signatures from cancer cell lines, however, the elastase MME was identified as a novel hypoxia-induced gene in lung cancer. Due to the lack of hypoxia effects on MME expression in NSCLC cell lines in contrast to carcinoma-associated fibroblasts, a direct up-regulation of stroma fibroblast MME expression under hypoxia might contribute to enhanced aggressiveness of hypoxic cancers.

## Background

Survival following diagnosis of non-small cell lung cancer (NSCLC) is poor despite therapy [1]. Hypoxia is typically present in solid tumors like lung cancer and is known to enhance tumor progression and therapy resistance [2]. The effects of hypoxia are largely mediated by the hypoxia-inducible factors (HIFs) HIF-1α [3,4] and HIF-2α [5]. HIFs induce the expression of many different proteins that are involved in key functions of cancer cells, including cell survival, metabolic reprogramming, angiogenesis, invasion, and metastasis. Under normoxic conditions, HIFs are rapidly degraded, while under hypoxia they are stabilized [3,4]. In addition to oxygen-dependent regulation, HIFs can be up-regulated by other mechanisms, e.g. growth factor induced pathways [3,4]. The biological response of tumors to hypoxia is influenced by the interplay of neoplastic cancer cells and the surrounding stroma cells, e.g. cancer-associated fibroblasts (CAFs) [6]. *Ex vivo* human cancer models based on the short-term culture of small tumor fragments or slices are suitable to study tumor responses within the natural *in situ* microenvironment, comprising a close contact between tumor cells and the accompanying stroma cells. Such models have been used e.g. for the study of drug effects in lung cancer [7] and other cancers [8,9]. Here we used a human *ex vivo* lung cancer model involving culture of fresh tumor fragments in a hypoxic atmosphere to mimic *in vivo* tumor hypoxia and performed a comparative expression profiling study. We found that hypoxia led to overexpression of a stem-cell marker with elastase activity, membrane metallo-endopeptidase (MME), in tumor fragments, which was attributable to carcinoma-associated fibroblasts, not the neoplastic cancer cells.

## Methods

### Lung cancer fragments

Tumor tissue samples from 70 consecutive patients with NSCLC who were referred for surgical resection to the Division of Thoracic and Hyperbaric Surgery, Medical University of Graz, from May 2007 to May 2013, were included in the study. Patients with pre-operative chemotherapy were excluded from the study. Surgical specimens were dissected into small fragments using a razor blade and fragments were incubated in 35 mm Petri dishes (up to ten fragments per well) in 2 ml of DMEM/F-12 growth medium (Gibco, Carlsbad, CA) containing 10% fetal calf serum (Biowest Ltd, Ringmer, UK), 2 mM L-glutamine (Gibco), 100 U/ml penicillin, and 100 μg/ml streptomycin (Gibco). The study protocol was approved by the ethics review board of the Medical University of Graz. Signed informed consent was obtained from all patients prior to surgery.

### Cells

The human NSCLC cell lines A549 and A427 were purchased from Cell Lines Service (Eppelheim, Germany) and cultured in DMEM/F-12 medium containing the supplements described above. The human NSCLC cell lines NCI-H23, NCI-H358, NCI-H1299, and NCI-H441 were purchased from American Type Culture Collection (ATCC, Manassas, VA) and cultured in RPMI (Gibco), supplemented with 10% fetal calf serum (Biowest) and antibiotics.

Carcinoma-associated fibroblasts (CAFs) were isolated from three fresh NSCLC samples as described [10] and cultured in DMEM supplemented with 10% fetal calf serum (Biowest) and antibiotics. CAFs were identified to be positive for vimentin and negative for cytokeratin using immunofluorescence. The purity of the cells was 97-99%. Human lung fibroblasts were cultured from donor lungs that could not be used for transplantation as previously described [11].

### Hypoxic culture

Fragments were cultured for three days at 37°C in ambient (21%) oxygen or 1% oxygen in the automated Xvivo System G300CL (BioSpherix, Lacona, NY). NSCLC cells or fibroblasts were plated into cell culture flasks at 13,000/cm^2^ and let attach, thereafter cells were cultured for three days in ambient oxygen or 1% oxygen as described above. Exposure to oxygen was controlled throughout the experiments in the hypoxic workstation.

### MTT assay

The MTT assay (Chemicon, Billerica, MA) was performed on cultured fragments according to the manufacturer’s instructions. Briefly fragments were incubated in the MTT substrate solution for one hour and formazan was dissolved in isopropanol. After dissolving the formazan 100 μL of sample was analyzed on a colorimetric microplate reader at 570 nm. A549 cells were used as a positive control.

### Pimonidazole assay

The assay (Hypoxyprobe™, HPI, Burlington, MA) was performed essentially according to the manufacturer’s instructions. Fragments were incubated for one or three days in hypoxia or normoxia. Thereafter fragments were treated with 100 μM pimonidazole HCl (HPI) in hypoxia in the closed Xvivo hypoxic working chamber (BioSpherix) or in normoxia and incubated for one hour, fixed and paraffin embedded. Bound pimonidazole was visualized using mouse monoclonal pimonidazole antibody (1:50 dilution, HPI).

### RNA extraction and cDNA synthesis

Total RNA was extracted using the Qiagen RNeasy Mini kit (Qiagen, Hilden, Germany) and DNase digestion (Qiagen) according to the manufacturer’s instructions. RNA integrity was assessed using the Agilent 2100 Bioanalyzer and the Agilent RNA 6000 Nano Kit (Agilent, Palo Alto, CA). All samples exhibited a RIN (RNA Integrity Number) >5. Samples with RIN > 8 were eligible for microarray analysis. Total RNA (1 μg) was reverse transcribed using the RevertAid H Minus First Strand cDNA synthesis kit (Fermentas, Burlington, Canada).

### Quantitative real-time PCR

For single gene quantitative polymerase chain reactions (PCR) the 7900 Real-Time PCR System (Applied Biosystems, Foster City, CA) was used. Gene expression assays (TaqMan^®^ Gene Expression Assays, Applied Biosystems) suitable for this system were used for the detection of carbonic anhydrase IX, PPP1R3C, MME, KCTD11, FAM115C, and hexokinase 2. ACTB (ß-actin) was used as a reference gene. Primer data are indicated in Additional file 1: Table S2. The PCR was performed in 10 μl reactions containing cDNA (equal to 2.5 ng or 12.5 ng total RNA), 1× TaqMan^®^ Gene Expression Mastermix (Applied Biosystems) and 1× TaqMan^®^ Gene Expression Assay (Applied Biosystems). The mean threshold cycle (Ct) number of triplicate runs was used for data analysis. ΔCt was calculated by subtracting the Ct number of the gene of interest from that of the reference gene β-actin (ACTB). For calculation of differences between two groups, ∆Ct-values of the control group (normoxia) were substracted from ∆Ct-values of the treated group (hypoxia).

### Expression profiling

The microarray analysis was performed using GeneChip Human Gene 1.0 ST Arrays (Affymetrix, Santa Clara, CA). Manufacturer’s instructions were followed for the hybridization, washing, and scanning steps. Pre-labelled spike-in controls, unlabelled spike controls, and background probes were included in the analysis. All the microarray data are available at Gene Expression Omnibus (GEO; http://www.ncbi.nlm.nih.gov/geo/; accession number GSE30979).

### Processing of microarray data

Statistical analysis of the microarray data was performed using Partek Genomic Suite Software (Partek, St. Louis, MO). RMA (Robust Multi Chip Analysis) background correction of raw microarray data and normalization of expression values were performed using Partek Genomic Suite Software (Partek). Fold-changes of expression values were calculated as the ratio of the mean RMA corrected expression value in the hypoxic group to the normoxic group. Fold-change values <1 were converted to the negative of the inverse ratio. Hypoxic and normoxic samples were compared using the paired Student’s t-test. The false discovery rate (FDR) was set to 5% to correct for multiple testing. In the case of subgroup analyses, the threshold was set to *P*<0.005. A gene was considered modulated when at least one of the corresponding probe sets showed significantly different expression levels after correction for multiple testing with a minimal two-fold change.

### Meta-analysis of lung cancer transcriptome studies

Expression values for the genes of interest were obtained from four eligible lung cancer datasets published at Gene Expression Omnibus (GEO; http://www.ncbi.nlm.nih.gov/geo/). Details on data processing and patient characteristics are reported at GEO and in the cited literature. Details on data retrieval are indicated in Additional file 1.

### Statistical analysis

Meta-analysis of the effect of MME on patient survival after surgery was performed with a proportional hazards model with Gaussian random effects [12,13] using the package coxme 2.1-3 of R 2.13.2 statistical software (http://www.r-project.org). For details see Additional file 1. All other data were compiled and analyzed with the SPSS software package, version 18.0 (Chicago, IL). Group differences were calculated with the paired Student’s t-test, one-sample Student’s t-test, Mann–Whitney-U test, or Wilcoxon signed rank test as applicable. *P*-values smaller than 0.05 were considered significant.

## Results

### Apoptosis and hypoxia markers in NSCLC fragments

**Figure 1:**
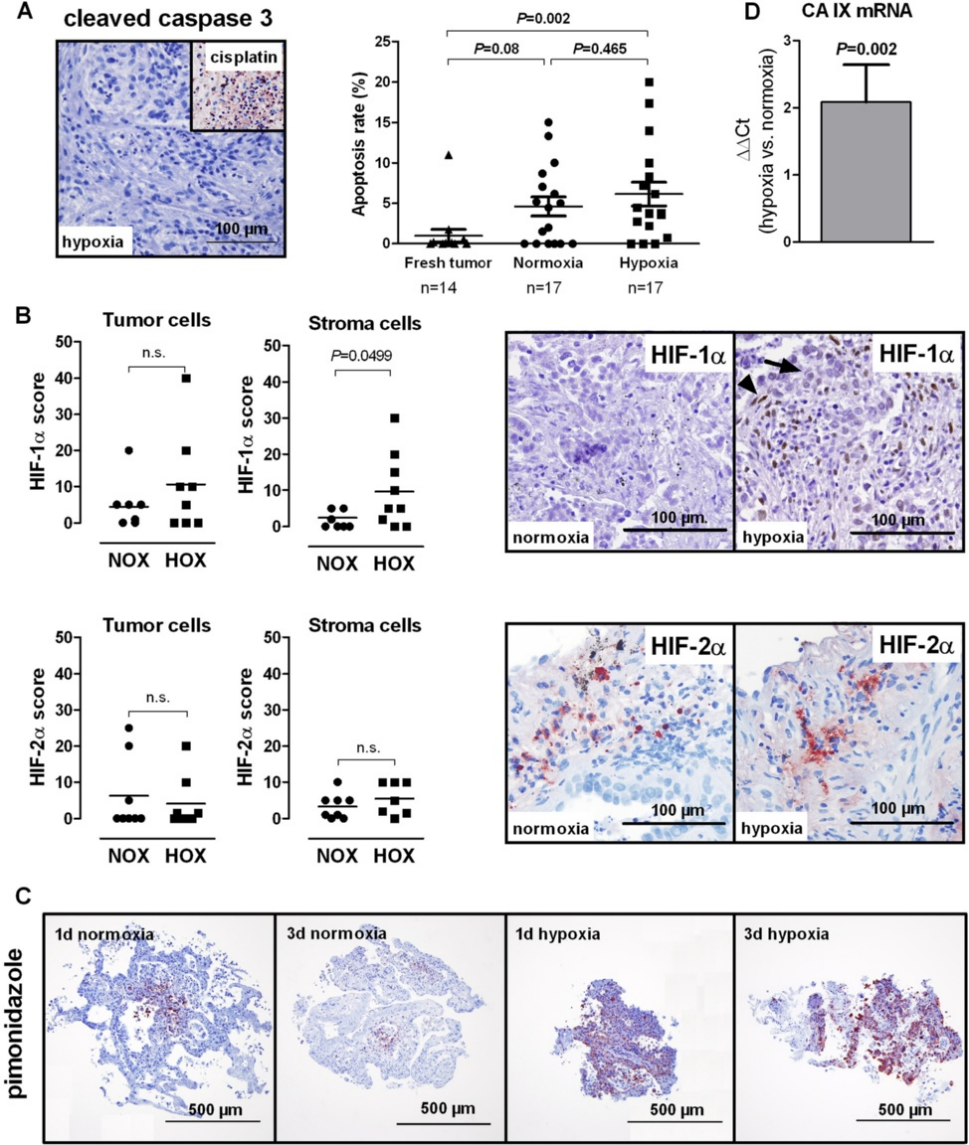
**Apoptosis and hypoxia markers in normoxic and hypoxic *ex vivo *cultured lung cancer fragments. (A)** Apoptosis is not increased in hypoxic *ex vivo* cultured NSCLC fragments. Representative images of cleaved caspase 3 staining for the assessment of apoptosis are shown: a hypoxic adenocarcinoma fragment cultured for three days in hypoxia without visible apoptosis and a fragment treated with 32 μM cisplatin as positive control. Right, apoptosis rates were determined by counting cleaved caspase 3 positive tumor cells in a blinded manner. Lines indicate mean +/- SEM. Groups were compared with the Mann–Whitney-U test. **(B)** HIF-1α and HIF-2α immunoreactivity in NSCLC fragments after three days of incubation in hypoxia or normoxia. Arrowhead: stroma cell, arrow: tumor cell. HIFs were evaluated in tumor cells and stroma cells semi-quantitatively in a blinded manner. Groups were compared with the paired Student’s t-test. **(C)** Pimonidazole staining indicating hypoxia in fragments cultured for one or three days in hypoxia or normoxia. Representative images are shown. All sections were counterstained with hematoxylin. **(D)** Carbonic anhydrase IX (CA IX) mRNA in hypoxic vs. normoxic NSCLC fragments (n = 14 patients). No difference in expression gives a value of zero. Significance was calculated with the single group Student’s t-test. Results are mean +/- SEM. NOX, normoxia; HOX, hypoxia.

NSCLC tissue was fragmented immediately after surgery. Fragments were maintained in culture medium for three days, both in ambient oxygen or hypoxia (1% O_2_). The largest diameter measured from paraffin sections (n = 430) was 1.19 mm (median, range 0.2 mm to 2.9 mm), the smallest diameter was 0.8 mm (median, range 0.2 mm to 2.2 mm). There was no significant difference between the size of fragments cultured in normoxia or hypoxia (*P* = 0.972). The histomorphology of cultured NSCLC fragments resembled the growth patterns usually found in freshly resected NSCLC tissue. Cancer cell nests were found in close proximity to stroma-rich regions with only scattered tumor cells. Tumor cells were found in the vast majority of cultured fragments, large necroses were rare. The MTT assay was used to determine, whether cells in cultured fragments were metabolically active, as an indirect qualitative indicator of cell viability. All fragments tested (n = 15 hypoxic and 15 normoxic fragments from three different patients) showed a positive MTT reaction. Apoptosis rates of tumor cells were investigated using immunohistochemical staining for cleaved caspase 3 (Figure 1A). No significant difference was found between apoptosis rates in normoxic and hypoxic fragments (Figure 1A).

HIF-1α and HIF-2α immunohistochemistry was performed in NSCLC fragments cultured for three days under normoxia or hypoxia (Figure 1B). HIF-1α was localized predominantly in the nucleus, while HIF-2α was found in the cytoplasm. Both, cytoplasmic and nuclear localization of HIF-1α and HIF-2α, have been reported [14,15]. Hypoxic fragments displayed more pronounced staining for HIF-1α than normoxic fragments, though the difference was significant only in stroma cells, not in tumor cells (Figure 1B). For HIF-2α no difference between fragments cultured in hypoxia or normoxia was found, neither in tumor cells, nor in stroma cells (Figure 1B). Next we assessed the presence of hypoxia in cultured fragments using pimonidazole. Figure 1C shows examples of NSCLC fragments cultured in normoxia or hypoxia for one and three days. Pimonidazole was bound almost to the entire hypoxic fragments, while only focal pimonidazole binding occurred in normoxic fragments, obviously due to diminished oxygen concentrations in central fragment areas. In several hypoxic fragments some cells showed higher pimonidazole binding than others (Figure 1C), which might be caused by a different content of redox enzymes (J. Raleigh, Hypoxyprobe Inc. and UNC School of Medicine, Chapel Hill, North Carolina, USA, personal communication) or due to other cell-related causes, such as differences in pimonidazole uptake or pH [16]. Expression of the HIF-1α target carbonic anhydrase IX (CA IX), which was shown to be linked to hypoxia in NSCLCs *in vivo*[17], was analyzed by quantitative PCR. CA IX mRNA levels were significantly higher in hypoxic fragments compared to normoxic fragments (Figure 1D). Taken together, NSCLC fragments remained viable for the duration of the experiments and hypoxia markers were increased under hypoxic treatment.

### Gene regulation by hypoxia in NSCLC fragments

**Figure 2:**
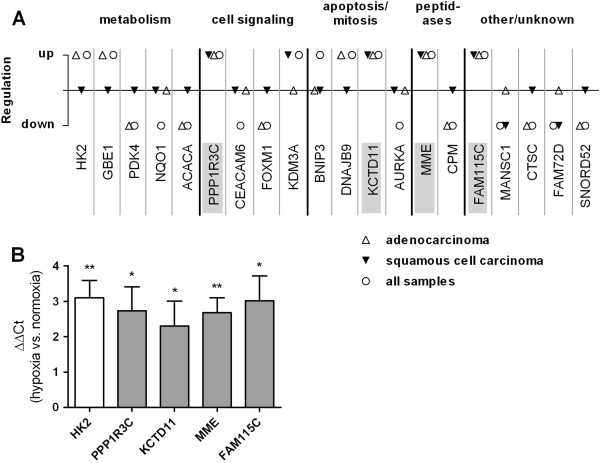
**Top 20 hypoxia-regulated genes according to *P*-values and validation of microarray results using quantitative PCR. (A)** Only significantly regulated genes with a minimum fold-change of 2 are included. The analysis was performed for all samples (n = 20 patients) or for adenocarcinoma (n = 10 patients) and squamous cell carcinoma (n = 10 patients) separately. Overlapping genes, regulated in the same direction in both histological subtypes are highlighted. **(B)** Confirmation of the up-regulation of overlapping hypoxia-regulated genes in hypoxic and normoxic fragments derived from NSCLC surgical specimens from four patients by quantitative PCR. Results are shown as mean +/-SEM. No difference in expression gives a value of zero. Significance was calculated with one-sample Student’s t-test. HK2, hexokinase 2; MME, membrane metallo-endopeptidase; KCTD11, potassium channel tetramerisation domain containing 11; PPP1R3C, protein phosphatase 1 regulatory subunit 3C; FAM115C, family with sequence similarity 115 member C. *P < 0.05, **P < 0.01.

In order to identify hypoxia-responsive genes, normoxic and hypoxic fragments derived from ten patients were subjected to expression profiling. A total of 107 genes were significantly regulated by hypoxia; 28 genes were up-regulated (Table 1) and 79 genes were down-regulated (Additional file 2: Table S3). Hypoxia expression patterns differed between histological subtypes (Figure 2A). Four genes were significantly regulated in the same direction (up-regulated) in both subtypes with a minimal two-fold change: PPP1R3C (protein phosphatase 1 regulatory subunit 3C), KCTD11 (potassium channel tetramerisation domain containing 11), FAM115C (family with sequence similarity 115 member C), and membrane metallo-endopeptidase (MME, CD10, neutral endopeptidase, neprilysin) (Figure 2A). The GO annotations (http://www.geneontology.org) for the gene products are as follows: PPP1R3C, regulation of glycogen biosynthesis; KCTD11, regulation of cell proliferation; and MME, proteolysis. The gene product of FAM115C has unknown function. Hypoxia-regulation of the four overlapping hypoxia genes and of the known hypoxia-responsive gene hexokinase 2 (HK2) was confirmed using real-time PCR in normoxic and hypoxic fragments from an independent validation set (n = 8, Figure 2B).

**Table 1:** Genes up-regulated by hypoxia

**Gene symbol**	**Gene name**	***P***	**Fold-change**
PPP1R3C	Protein phosphatase 1, regulatory (inhibitor) subunit 3C	7,6E-07	2,5
HK2	Hexokinase 2	3,4E-06	2,1
GBE1	Glucan (1,4-alpha-), branching enzyme 1	3,9E-06	2,3
KDM3A	Lysine (K)-specific demethylase 3A	5,6E-06	2,0
KCTD11	Potassium channel tetramerisation domain containing 11	6,3E-06	2,2
FAM115C	Family with sequence similarity 115, member C	1,1E-05	2,3
BNIP3	BCL2/adenovirus E1B 19 kDa interacting protein 3	3,6E-05	2,1
DNAJB9	DnaJ (Hsp40) homolog, subfamily B, member 9	4,6E-05	2,4
MME	Membrane metallo-endopeptidase	7,3E-05	2,5
IL8	Interleukin 8	8,3E-05	2,3
DTNA	Dystrobrevin, alpha	9,0E-05	2,1
C7orf68	Chromosome 7 open reading frame 68	9,5E-05	2,4
LOX	Lysyl oxidase	1,2E-04	2,7
HSPA13	Heat shock protein 70 kDa family, member 13	1,7E-04	2,2
LRRC49	Leucine rich repeat containing 49	1,9E-04	2,0
STC2	Stanniocalcin 2	2,2E-04	2,2
TREM1	Triggering receptor expressed on myeloid cells 1	2,5E-04	2,3
CDR1	Cerebellar degeneration-related protein 1	2,5E-04	2,2
ADM	Adrenomedullin	2,5E-04	2,2
NUCB2	Nucleobindin 2	3,6E-04	2,4
TSPYL2	TSPY-like 2	5,8E-04	2,0
DDIT3	DNA-damage-inducible transcript 3	6,3E-04	2,5
C1orf129	Chromosome 1 open reading frame 129	8,2E-04	3,4
FICD	FIC domain containing	1,0E-03	2,4
FABP3	Fatty acid binding protein 3	1,3E-03	2,0
ABI3BP	ABI family, member 3 (NESH) binding protein	1,3E-03	2,0
NOG	Noggin	1,4E-03	2,1
SEC11C	SEC11 homolog C	4,0E-03	2,2

Interestingly, the overall impact of hypoxia on gene expression was lower than the impact of histology or inter-patient variability (Additional file 3: Figure S1). Normoxic and hypoxic fragments derived from each patient clustered together significantly in 9 of 10 patients in pvclust analysis (Additional file 3: Figure S1). Both clusters on the top of the hierarchy were significant in pvclust analysis. One cluster contained four squamous cell carcinomas, the other cluster contained all adenocarcinomas and one squamous cell carcinoma (Additional file 3: Figure S1).

### MME immunohistochemistry

**Figure 3:**
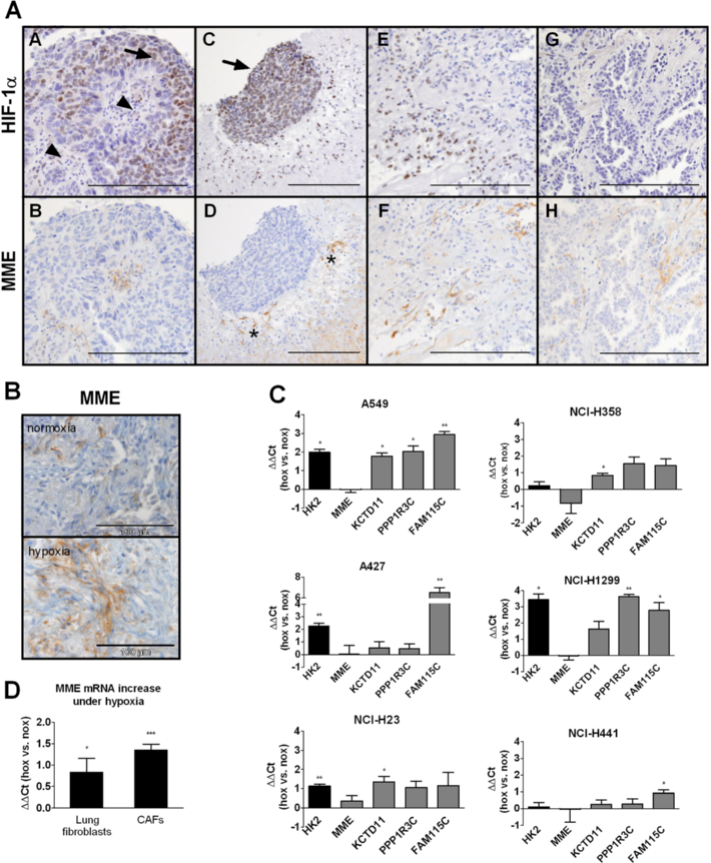
**Expression of MME in NSCLC samples, NSCLC cell lines, and fibroblasts. (A)** MME and HIF-1α were stained in consecutive sections of fresh NSCLC. Representative areas from a sample with high HIF-1α staining in tumor (arrow) and stroma cells (arrowhead) are shown (images A-F). While MME positive stroma cells were found predominantly in HIF-1α positive areas, no association of HIF-1α and MME was found in tumor cells. Note the intensely MME positive stroma cells (asterix) surrounding an islet of tumor cells, which were MME negative. Images G and H show a sample from a different patient. In this patient MME staining in stroma cells was apparently unrelated to HIF-1α. Scale bar: 200 μm. **(B)** Immunohistochemistry for MME in normoxic and hypoxic fragments from a single patient. **(C)** NSCLC cells were cultured in hypoxia (1% oxygen) or ambient oxygen for three days and mRNA levels of hexokinase 2 and of the four overlapping hypoxia genes were analyzed. Expression levels in hypoxia relative to normoxia are shown. Results are mean +/- SEM from three independent experiments. **(D)** Human lung fibroblasts from three different donors and carcinoma-associated fibroblasts (CAFs) isolated from NSCLC from three different patients were cultured in hypoxia (1% oxygen) or ambient oxygen for three days and MME mRNA levels were analyzed. Results are mean +/- SEM from n = 5 to 7 independent experiments. **(C and D)** Groups were compared with one-sample Student’s t-test. HK2, hexokinase 2; MME, membrane metallo-endopeptidase; KCTD11, potassium channel tetramerisation domain containing 11; PPP1R3C, protein phosphatase 1 regulatory subunit 3C; FAM115C, family with sequence similarity 115 member C; nox, normoxia; hox, hypoxia. *P < 0.05, **P < 0.01, ***P < 0.001.

In order to determine the cell types responsible for MME expression in our model we performed immunohistochemical staining in fresh NSCLC specimens from 12 patients. MME-positive neoplastic tumor cells were found in 80% and scattered MME-positive stroma cells were found in 54% of fresh cancer specimens. Up to 30% of stroma cells were MME positive in cultured fragments, indicating generally increased MME expression in tumor stroma cells under stress conditions (Figure 3B). Using this technique, no difference in MME staining in normoxia or hypoxia was found. However, since immunohistochemistry is a semiquantitative method, only large differences in expression levels can be detected. Next, consecutive sections of fresh NSCLC samples from 30 patients were stained for MME and HIF-1α in order to analyze, whether the expression of both is linked *in vivo.* Similar to the first series MME staining was found in tumor cells in 21/30 samples (70%) and in stroma cells in 10/30 samples (33.3%; 5 to 20% of stroma cells were MME positive). In 8/30 patients (26.7%), HIF-1α positivity was found in tumor cells. In 2/30 (6.7%) patients also stroma cells were HIF-1α positive. In a sample with very high stroma and tumor cell HIF-1α expression, HIF-1α and MME staining overlapped in stroma cells, but not in tumor cells (Figure 3A, images A-F). On the other hand in another patient with MME stroma staining no HIF-1α was found (Figure 3A, images G and H). In tumor cells MME and HIF-1α staining were not strongly related. Together this indicated to us that in some patients hypoxia may be linked to MME expression in the tumor stroma.

### Expression of hypoxia-regulated genes in NSCLC cells and carcinoma-associated fibroblasts (CAFs)

We further analyzed the expression of MME under hypoxia in both, NSCLC cell lines and fibroblasts, which are the predominant cell type in lung cancer stroma, using quantitative PCR. PPP1R3C, KCTD11, FAM115C, and HK2, a well-known hypoxia-regulated gene, were up-regulated by hypoxia in a panel of NSCLC cell lines to variable degrees, while MME mRNA showed no increase in expression under hypoxia in any of the cell lines (Figure 3C). On the contrary in carcinoma-associated fibroblasts (CAFs) from NSCLC and, to a lesser extent, in primary lung fibroblasts MME mRNA was significantly up-regulated by hypoxia (Figure 3D).

### MME expression is an adverse prognostic factor in lung adenocarcinoma patients

Next, we examined whether expression of the four hypoxia genes was associated with survival in patients with NSCLC. Due to the relatively short observation period in our patient cohort, we used large published microarray datasets containing gene expression data linked to clinical and prognostic information in NSCLC patients. The Gene Expression Omnibus (GEO; http://www.ncbi.nlm.nih.gov/geo/) is one of the largest microarray databases. A search for GEO datasets/series using the search criteria „lung cancer 50:500[Number of Samples]” yielded 84 results (status June 2011). Of these 84 datasets/series, 68 contained expression profiling data. Four of these series included expression data of a minimal number of 50 NSCLC patients treated by surgery with linked information on survival, GSE11969 [18], GSE13213 [19], GSE14814 [20], and GSE19188 [21]. Altogether 342 patients were included in the meta-analysis.

**Figure 4:**
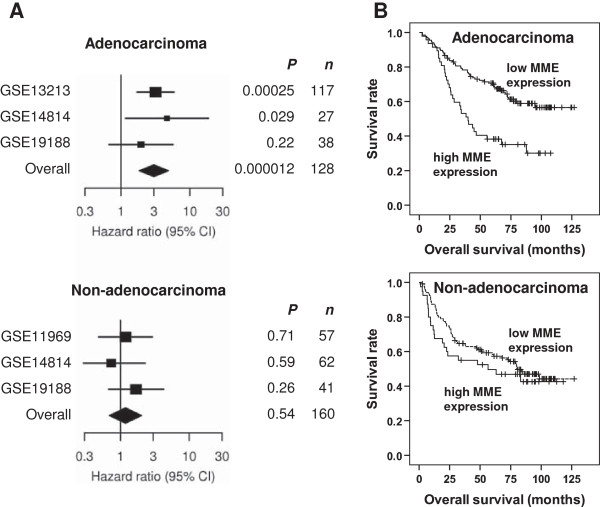
**MME expression is associated with poor prognosis in lung adenocarcinoma patients treated with surgery. (A)** Based on the expression of MME in microarrays from tumor specimens, NSCLC patients from publically available GEO microarray series were stratified into patients with high MME expression (the highest quartile) versus low MME expression (the remaining three quartiles). The association between MME expression and overall survival was calculated within the GEO series, and in cohorts derived by combining the different GEO series. All analyses were performed in a multivariate manner with pathological tumor stage as stratification variable. Adenocarcinoma and non-adenocarcinoma patients from each study were analyzed separately. Study GSE13213 contained only adenocarcinoma patients. From study GSE11969 only the non-adenocarcinoma patients were included due to potential overlap with patients from GSE13213, who were operated at the same centre. Results are displayed as hazard ratio for death in the high MME group versus the low MME group +/- 95% confidence interval. **(B)** Kaplan-Meier plot of overall survival in adenocarcinoma patients (n = 182) and non-adenocarcinoma patients (n = 160) from the combined cohort dichotomized according to the expression levels of MME.

Of the four overlapping hypoxia genes MME was the only prognostic factor for overall survival (*P* = 0.00057) in a multivariate analysis with pathological tumor stage as stratification variable. The interaction between MME and histology (adenocarcinoma vs. non-adenocarcinoma) was statistically significant (*P* = 0.027). Thus survival analyses were performed in adenocarcinoma patients and non-adenocarcinoma patients separately (Figure 4). High expression of MME was significantly associated with poorer survival in adenocarcinoma patients of series GSE13213 (*P* = 0.00025) and series GSE14814 (*P* = 0.029), and in the combined cohort including 182 patients (*P* = 0.000012, Figure 4A,B). In series GSE13213 and in the combined cohort, but not in series GSE14814, the association between MME and survival was significant even after Bonferroni correction for multiple testing for all genes/probe sets in all the studies. In the combined cohort of adenocarcinoma patients the hazard ratio (HR) for death in the high MME group was 3.0 (95% CI 1.83- 4.90; Figure 4A). In non-adenocarcinoma patients the risk for death was not different in the high MME group compared with the low MME group (HR = 0.93, 95% CI 0.35- 2.4, *P* = 0.496; Figure 4A,B).

## Discussion

Identifying hypoxia-regulated genes may promote understanding of the molecular response to hypoxic stress in cancers. Changes in gene expression in hypoxic cancer cells have been studied extensively *in vitro*. However, hypoxia-responses *in vivo* may differ from the *in vitro* situation due to the complex tumor microenvironment. In fact, hypoxia activates tumor promoting stroma cells and HIF-1α has been identified as the major driver of tumor-stroma “co-evolution” [22]. Here we studied hypoxia-induced gene expression experimentally in human cancer tissue in its preserved 3D-structure. In this fragment model the tissue contains both tumor and stroma cells and mimics the *in vivo* situation.

The model has several advantages compared to *in vitro* cancer cell lines. The tumor cells remain in contact with their original tumor microenvironment (stroma cells, extracellular matrix), the 3D-morphology is preserved, and inter-patient variability is taken into account by using material derived from different patients. The major limitation of our study is that the exact oxygen concentration could only be controlled on the surface of the fragments. Inside the tumor fragments there are supposed to be oxygen gradients, depending on the size and composition of the tissue fragment. The size of normoxic and hypoxic fragments did not differ in our study. In fact, using pimonidazole staining, fragments cultured in hypoxia were found to be entirely hypoxic, while only a core of hypoxia was found in fragments cultured in normoxia. In addition to pimonidazole, also other major hypoxia-markers were significantly increased in the hypoxic fragments, such as HIF-1α and CA IX. However, HIF-2α, which is known to be stabilized by hypoxia similarly to HIF-1α, was expressed only at low levels, both in normoxia and hypoxia, and was not elevated in hypoxic fragments. Different co-activators and different kinetics of activation under hypoxia [23] might play a role. This indicates that the difference in oxygen concentration was preserved despite the expected oxygen gradients inside the fragments. Furthermore the oxygen decline is supposed to occur in both, normoxic and hypoxic fragments. Thus our approach is feasible to study differential gene expression under high and low oxygen concentrations.

Apoptosis rates were comparable in NSCLC fragments cultured in 1% O_2_ or normoxia for three days. This agrees with our previous study where we showed that hypoxia-induced adaptation and cisplatin-resistance are reversible in lung cancer cells and occur without hypoxia-induced cell death and selection [24]. In an attempt to identify common hypoxia-regulated genes, Ortiz-Barahona et al. [25] identified 17 genes consistently up-regulated by hypoxia, hypoxia-mimetics, or HIF-1α using a meta-analysis of expression data from 16 GEO datasets. Of these 17, mostly well-known hypoxia-regulated genes, 65% appear among the significantly regulated genes in our study (after correction for multiple testing). When we compared a hypoxia signature found to be prognostically relevant in many cancers (the “hypoxia metagene” [26]) with our hypoxia profile, we also found a considerable overlap. Approximately half of the top-ranked hypoxia-induced genes with prognostic relevance identified by Buffa et al. [26] were significantly up-regulated by hypoxia in our study.

Four genes were significantly up-regulated by hypoxia in both adenocarcinoma and squamous cell carcinoma fragments in our setting. We confirmed the differential expression of the four overlapping hypoxia genes under hypoxia in an independent validation set using quantitative PCR (qPCR). Also the well-established hypoxia-responsive gene HK2, which phosphorylates glucose and thus contributes to the glycolytic flux in cancer cells, was significantly up-regulated by hypoxia in the fragments, both in the microarray analysis and by qPCR.

The four hypoxia-genes identified in our study have been found to be up-regulated by hypoxia in several microarray studies, however these findings were not validated e.g. by qPCR [27-32]. To the best of our knowledge, validated data on hypoxia-regulation of the four hypoxia-regulated genes exist for PPP1R3C (up-regulated in MCF-7 breast cancer cells) [33] and on MME, which was shown to be up-regulated in primary rat astrocytes [34] and down-regulated in pulmonary artery smooth muscle cells [35], human neuroblastoma cells [34], rat neurons [34], and mouse neurons [36]. Cobalt chloride, a hypoxia mimetic, was shown to reduce MME expression in prostate cancer cell lines [37], and human umbilical vein endothelial cells [37]. In addition, exposure of rats and mice to a hypoxic atmosphere led to down-regulation of MME expression [38,39].

In our study we found MME localized to neoplastic tumor cells, but also to stroma cells in fresh NSCLC tissue, which is in line with published data [40,41]. The observed up-regulation of MME under hypoxia in NSCLC fragments might thus be attributable to tumor cells or stroma cells, or both. While the hypoxic regulation of KCTD11, FAM115C, PPP1R3C and HK2 was also observed to a variable degree in a panel of NSCLC cell lines cultured as a monolayer, MME was not regulated by hypoxia in the cell lines in our study. Fibroblasts are the predominant cell type in lung cancer stroma [42]. When we studied MME mRNA in CAFs we found a significant induction by hypoxia. A similar effect was found in normal lung fibroblasts, however to a lesser extent. The exact mechanism of MME regulation by hypoxia in fibroblasts remains to be elucidated. The proximal promoter regions of the different MME splice variants have been shown to harbour binding sites for the transcription factors Sp1, PEA3 and PU.1 [43]. PEA3 (also known as E1AF and ETV4) is a member of the Ets-family of transcription factors. PEA3 was shown enhance cancer metastasis [44]. Recently, PEA3 has been shown to interact with HIF-1α [45]. This might at least partially be responsible for the observed effect of hypoxia on MME expression.

MME, which is identical to common acute leukemia antigen (CALLA), is a 90–110 kDa zinc binding cell surface peptidase, which cleaves small peptides, such as atrial natriuretic peptide, substance P, endothelin-1, and bombesin (for review see [46,47]). It also possesses elastase activity [48]. MME is a membrane-bound protein, however, as was recently shown, MME can be released to the microenvironment of cells in exosomes [49]. MME is expressed in a variety of non-malignant and malignant tissues (for review see [46,47]) including lung cancer [40,50-52]. In small-cell lung carcinoma (SCLC) cells, bombesin-like peptides, substrates for MME, are autocrine growth factors. Cleaving these peptides by recombinant MME has been shown to inhibit SCLC cell proliferation [53,54]. In NSCLC cells, recombinant MME inhibited tumor cell proliferation *in vitro*, but only at very high concentrations and after long exposure [54]. On the contrary, MME inhibitors have been found to decrease cell proliferation in the airway wall in response to cigarette smoke in rats [55]. While the role of MME in neoplastic tumor cells is still unclear, several reports suggest that stroma cell MME expression plays a role in tumor progression. MME-positive stroma cells, including mesenchymal stem cells and fibroblasts, have been shown to promote tumor aggressiveness and metastasis [56,57]. Elastin is degraded by MME [48], which might facilitate tumor and/or stroma cell invasion.

In order to analyze, whether levels of the common hypoxia-genes identified in our study are associated with overall survival in NSCLC patients we used all eligible studies deposited in one of the largest microarray depositories, the GEO database. We were able to show that MME expression is a highly significant, independent adverse prognostic factor in surgically treated lung adenocarcinoma patients in multivariate analysis involving tumor stage and MME status. No association was found in the subgroup of non-adenocarcinoma patients. The reason for the different results in the histological subgroups is unknown, however, lung adenocarcinomas have been shown to possess more elastin than squamous cell carcinomas [58]. Since the largest study with 116 adenocarcinoma patients (GSE13213) contained only adenocarcinomas, a study-bias cannot be excluded.

To the best of our knowledge, three other studies examined the association of MME expression and survival in lung cancer [40,41,51]. All studies are immunohistochemical studies. In a study by Kristiansen et al. [51] in 114 NSCLC patients no association of MME immunostaining and survival was found. Only neoplastic cancer cells were evaluated in that study (G. Kristiansen, personal communication). In a recent study by Ono et al. [41] on 142 stage I squamous cell lung carcinoma patients MME expression was examined in tumor cells and stroma cells separately. Patients with low MME expression in stroma or in tumor cells survived slightly longer, but the differences were not significant. In a study by Gurel et al. [40] MME expression was studied in tumor cells and stroma cells in 66 patients with NSCLC using immunohistochemistry. In the squamous cell carcinoma subgroup high tumor cell and stroma cell MME expression were both associated with poor overall survival. In non-squamous cell NSCLC (35 patients, adenocarcinoma, large cell carcinoma, sarcomatoid carcinoma, and mixed types) the opposite association was found. No stroma cell MME expression was found in that subgroup [40]. The low number of patients may make the interpretation of these results difficult.

All of these studies were immunohistochemical studies, in contrast to our MME mRNA based survival analysis. Since MME may be excreted in exosomes [49], which has been shown e.g. for mesenchymal stem cells [59], the question arises, whether MME was excreted and then lost during conventional tissue fixation and immunohistochemistry. With immunohistochemistry thus the extent of MME expression in cancer tissue may be underestimated. This is supported by the fact that CAFs isolated from all three NSCLCs expressed MME mRNA in our study, while in the studies mentioned above high MME staining in stroma cells was found only in 11% to 19% of cases. This underestimation may partly explain the lack of association between MME expression and worse prognosis in the mentioned studies, as opposed to our mRNA based study.

Additional studies examined the expression of MME in combination with other factors and survival. In the study by Tokuhara et al. [50] 132 NSCLC patients were grouped according to their tumor MME mRNA and aminopeptidase N mRNA expression. Patients assigned to the group with high MME and low aminopeptidase N mRNA showed significantly improved survival. No analysis on MME expression alone was performed. Tumor tissue samples were selected to contain primarily cancer cells in that study. In a study by Navab et al. [10] MME was among a subset of eleven genes identified to be up-regulated in cancer associated fibroblasts, forming a prognostic gene-expression signature in NSCLC.

## Conclusions

The novel *ex vivo* model allowed for the first time to analyze hypoxia-regulated gene expression in preserved human lung cancer tissue. The study shows that gene expression profiles in human hypoxic lung cancer tissue overlap with hypoxia-signatures from cancer cell lines, however, MME was identified as a novel hypoxia-induced gene in lung cancer. Despite the advantages of *ex vivo* tissue culture, cell monolayers still appear to be the method of choice to study mechanisms of adaptation of individual cell types to hypoxia, since the oxygen concentration can be controlled only on the surface of such three-dimensional structures. Thus we analyzed expression of the hypoxia-regulated genes identified in the NSCLC fragments in different NSCLC cell lines and primary CAFs isolated from NSCLC tissue. We show that MME expression is up-regulated by hypoxia in CAFs, not in NSCLC cells. High global levels of MME mRNA in NSCLC tissue were shown in our study to predict poor survival. A direct effect of hypoxia on stromal fibroblast MME expression might thus contribute to enhanced aggressiveness of hypoxic cancers.

## Abbreviations

NSCLC: Non-small cell lung cancer; MME: Membrane metallo-endopeptidase; CALLA: Common acute leukemia antigen; HIF: Hypoxia-inducible factor; CA IX: Carbonic anhydrase IX; ACTB: β-actin; FDR: False discovery rate; PPP1R3C: Protein phosphatase 1 regulatory subunit 3C; KCTD11: Potassium channel tetramerisation domain containing 11; FAM115C: Family with sequence similarity 115 member C; HK2: Hexokinase 2; CAFs: Carcinoma-associated fibroblasts; HR: Hazard ratio; CI: Confidence interval.

## Competing interests

The authors declare that they have no competing interests.

## Authors’ contributions

KL contributed to the study design and data interpretation, obtained funding for the project, carried out cell culture and pimonidazole experiments, performed RNA isolation and immunohistochemistry and prepared the manuscript. CW carried out experiments with NSCLC fragments. HHP and ES contributed to study design and data interpretation, contributed to immunohistochemistry analysis and revised the manuscript. JL and FM-SJ contributed to establishment of the fragment model, coordinated the isolation of fresh NSCLC fragments and revised the manuscript. NAH contributed to immunohistochemistry and revised the manuscript. BG and CG participated in the design of the project, performed the microarray analyses and participated in interpretation of the data. FQ contributed to the survival meta-analysis, performed the hierarchical cluster analysis, and revised the manuscript. PS provided donor lungs and revised the manuscript. SP provided clinical data of patients included in the meta-analysis and critically revised the manuscript. AH contributed to study design and project coordination and revised the manuscript. AO contributed to the study design and data interpretation. HO contributed to the study design and data interpretation, coordinated the project, obtained project funding and revised the manuscript. All authors read and approved the final manuscript.

## Pre-publication history

The pre-publication history for this paper can be accessed here:

http://www.biomedcentral.com/1471-2407/14/40/prepub

## Supplementary Material

Additional file 1 **Supplementary Methods. Table S1.** Probesets identifying genes of interest. **Table S2.** TaqMan^®^ Gene Expression Assays for qPCR. Click here for file

Additional file 2: Table S3 Genes down-regulated by hypoxia. Click here for file

Additional file 3: Figure S1 Hierarchical clustering of gene expression profiles including all genes. *P*-values were calculated with pvclust based on 1000 bootstrap replications in 1426 selected genes with highest variability. In this software *P*-values greater than 95% are considered significant. Bootstrap *P*-values (bp) are shown in green. Asymptotically unbiased *P*-values (au) are shown in red. Cluster numbers (edge #) are shown in grey. The most similar pairs of arrays (shortest dendrogram branches) were the hypoxic and normoxic fragments from each patient. This close similarity was significant in 9 of 10 patients. Ad, adenocarcinoma; Sq, squamous cell carcinoma; No, normoxia; Hy, hypoxia. Click here for file
