# Supplementary material for: Hypoxia increases membrane metallo-endopeptidase expression in a novel lung cancer ex vivo model – role of tumor stroma cells
Source: BMC Cancer. 2014 Jan 25;14:40. doi: 10.1186/1471-2407-14-40 (PMC3905926; doi:10.1186/1471-2407-14-40)
Supplement: Additional file 1 — Supplementary Methods. Table S1. Probesets identifying genes of interest. Table S2. TaqMan® Gene Expression Assays for qPCR. [file 1471-2407-14-40-S1.pdf]

Supplementary Methods

Immunohistochemistry

Briefly, tissue sections were deparaffinized and rehydrated according to standard protocols. The following antibodies were used: rabbit polyclonal antibody against human/mouse cleaved caspase 3 (1:100 dilution, R&D Systems, Minneapolis, MN), mouse monoclonal antibody against human HIF-1α (1:100 dilution, Becton Dickinson, Franklin Lakes, NJ), mouse monoclonal antibody against human HIF-2α (EP-190b, 1:50 dilution, Abcam, Cambridge, UK), mouse monoclonal antibody against human MME/CD10 (1:50 dilution, Novocastra, Wetzlar, Germany). Staining with primary antibodies was performed for one hour at room temperature. The primary antibody was detected with the Ultravision LP detection system (Thermo Scientific, Waltham, MA). Peroxidase activity was visualized by incubation with 3-amino-9-ethylcarbazole solution (Dako, Glostrup, Denmark). In the case of MME, CC1 buffer (Ventana, Strasbourg, France) was used for antigen retrieval and visualization of the primary antibody was achieved with the iVIEW detection system (Ventana). Counterstaining was performed with hematoxylin. As a negative control, isotype control antibodies were used as primary antibodies or staining was performed in the absence of the primary antibody.

Evaluation of immunohistochemistry

All of the sections were examined by a thoracic pathologist (E.S.) in a blinded manner at x400 magnification using a Zeiss Axioplan 2 microscope (Oberkochen, Germany). Staining scores were calculated by multiplying the percentage of positive cells by the staining intensity. Apoptosis rates were expressed as percentage of cleaved caspase 3 positive tumor cells among all tumor cells and were determined by examination of the entire stained fragments at x400 magnification. Images were taken using an Olympus BX51 microscope, a DP12 digital camera system and Cell^D software (Olympus, Hamburg, Germany).

Meta-analysis of MME and survival in NSCLC patients

The association between expression of the four hypoxia genes (probe sets corresponding to the genes) and overall survival of NSCLC patients was assessed in each of the GEO series separately and in a meta-analysis. While probe sets for PPP1R3C and MME were present on all the arrays used in the four studies, probe sets for FAM115C and KCTD11 were present only in two datasets, GSE13213 and GSE19188 (Supplementary Table 1). From the GSE11969 series only non-adenocarcinoma patients were included due to potential overlap with patients from GSE13213, who were operated at the same centre. The expression of MME significantly correlated between the respective probe sets in all studies (Pearson correlation coefficients 0.86 to 0.98), while in the case of all other genes with more than one probe set, only moderate to weak correlations were found, possibly due to the detection of different splice variants/isoforms by the different probe sets. Only the well correlated MME probe sets were averaged. In all other cases each individual probe set was used for survival analysis.

According to the expression levels of genes/probe sets, patients were dichotomized into the “high expression group” (comprising 25% of patients with the highest expression) and the “low expression group” (the remaining patients). Patients with overall survival shorter than one month after surgery were excluded from the analysis. All analyses were performed in a multivariate manner with pathological tumor stage as stratification variable. Survival analyses were compiled within individual studies using the Cox proportional hazards model with pathological tumor stage as stratification variable. Meta-analysis of the effect of MME on patient survival after surgery was performed with a proportional hazards model with Gaussian random effects using the package coxme 2.1-3 of R 2.13.2 statistical software (www.r-project.org). This software allows to specify normally distributed random effects within a proportional hazards model and to test them by the likelihood ratio test. Tumor stage, histological type and MME expression were available for every study. Due to the heterogeneity of survival distributions the analysis was stratified with respect to study and

tumor stage. MME, histological type and their interaction were included as fixed effects. In the meta-analysis the interaction of MME with study was included as random effect.

Hierarchical cluster analysis

All genes were included in hierarchical cluster analysis. In order to check stability the clustering was analyzed by multiscale bootstrap resampling (pvclust [1]). Due to limited computing rescources only 5% of the genes with the highest MAD (median absolute deviation, a robust measure of variability) were included (1426 genes) [2]. It was observed that the clustering was unchanged by the selection compared to clustering without selection (Partek software).

Immunofluorescence

Cancer-associated fibroblasts (CAFs) were fixed in methanol, permeabilized with 0.02 % Triton X-100 for 30 min and stained with rabbit antibody to vimentin (Cell Signaling Technology, Danvers, MA) or mouse antibody to cytokeratin (Dako, Glostrup, Denmark, clone MNF116) at 4℃ overnight. Alexa-594 conjugate d goat anti-mouse antibody (Invitrogen, Life Technologies, Paisley, United Kingdom) or Alexa-594 conjugated goat anti-rabbit antibody (Molecular Probes Life Technologies, Paisley, United Kingdom) were used as secondary antibodies, counterstaining was performed with DAPI (Vector laboratories, Burlingame, CA).

References

1. Suzuki R, Shimodaira H: **Pvclust: an R package for assessing the uncertainty in hierarchical clustering.** *Bioinformatics* 2006, **22**(12):1540-1542.
2. Remke M, Hielscher T, Korshunov A, Northcott PA, Bender S, Kool M, Westermann F, Benner A, Cin H, Ryzhova M, Sturm D, Witt H, Haag D, Toedt G, Wittmann A, Schottler A, von Bueren AO, von Deimling A, Rutkowski S, Scheurlen W, Kulozik AE, Taylor MD, Lichter P, Pfister SM: **FSTL5 is a marker of poor prognosis in non-WNT/non-SHH medulloblastoma.** *J Clin Oncol* 2011, **29**(29):3852-3861.

Supplementary Table 1. Probesets identifying genes of interest

GEO series	Array	Gene symbol / name			
		PPP1R3C	KCTD11	MME	FAM115C
GSE11969	Agilent Homo Sapiens 21.6K Custom Array	A_23_P35414	n.a.	A_23_P212061	n.a.
GSE13213	Agilent-014850 Whole Human Genome Microarray	A_23_P35414	A_23_P354027	A_23_P212061<br>A_24_P260101	A_23_P397978<br>A_32_P214925
GSE14814	Affymetrix Human Genome U133A Array	204284_at	n.a.	203434_s_at<br>203435_s_at	n.a.
GSE19188	Affymetrix Human Genome U133 Plus 2.0 Array	204284_at<br>240187_at	235857_at	203434_s_at<br>203435_s_at	239315_at<br>1564027_a_at<br>1564028_s_at

n.a., not applicable (no probeset corresponding to the respective gene was present on the microarray)

Supplementary Table 2. TaqMan® Gene Expression Assays for qPCR

Gene symbol	Gene name	Company	Assay number.	Amplicon length
CA9	Carbonic anyhdrase IX	Applied Bioysystems	Hs00154208_m1	78
PPP1R3C	Protein phosphatase 1, regulatory subunit 3C	Applied Bioysystems	Hs00193642_m1	61
MME	Membrane metallo-endopeptidase	Applied Bioysystems	Hs00153510_m1	89
KCTD11	Potassium channel tetramerization domain containing 11	Applied Bioysystems	Hs00327145_s1	145
FAM115C	Family with sequence similarity 115, member C	Applied Bioysystems	Hs04186295_m1	105
HK2	Hexokinase 2	Applied Bioysystems	Hs00606086_m1	149
ACTB	Beta-actin	Applied Bioysystems	Hs99999903_m1	171
