# Supplementary material for: Hypoxia increases membrane metallo-endopeptidase expression in a novel lung cancer ex vivo model – role of tumor stroma cells
Source: BMC Cancer. 2014 Jan 25;14:40. doi: 10.1186/1471-2407-14-40 (PMC3905926; doi:10.1186/1471-2407-14-40)
Supplement: Additional file 2: Table S3 — Genes down-regulated by hypoxia. [file 1471-2407-14-40-S2.pdf]

Supplementary Table 3. Genes down-regulated by hypoxia

Gene symbol	Gene name	*P*	FC	Gene symbol	Gene name	*P*	FC
PDK4	Pyruvate dehydrogenase kinase, isozyme 4	5,2E-06	-2,0	CASC5	Cancer susceptibility candidate 5	2,8E-04	-2,3
MANSC1	MANSC domain containing 1	5,6E-06	-2,3	PBK	PDZ binding kinase	2,9E-04	-2,1
CPM	Carboxypeptidase M	8,6E-06	-2,3	DLGAP5	Discs, large (Drosophila) homolog-associated protein 5	3,0E-04	-2,3
NQO1	NAD(P)H dehydrogenase, quinone 1	1,9E-05	-2,4	KIF11	Kinesin family member 11	3,0E-04	-2,3
CTSC	Cathepsin C	2,4E-05	-2,0	TOP2A	Topoisomerase (DNA) II alpha	3,0E-04	-3,3
FAM72D	Family with sequence similarity 72, member D	2,7E-05	-2,2	IGHD	Immunoglobulin heavy constant delta	3,3E-04	-3,1
SNORD52	Small nucleolar RNA, C/D box 52	3,0E-05	-2,0	LOC100293211	LOC100293211	3,3E-04	-3,3
CEACAM6	Carcinoembryonic antigen-related cell adhesion molecule 6	4,2E-05	-4,0	PLA2G7	Phospholipase A2, group VII	3,8E-04	-2,7
ACACA	Acetyl-Coenzyme A carboxylase alpha	5,2E-05	-2,0	DHCR24	24-dehydrocholesterol reductase	3,8E-04	-2,6
AURKA	Aurora kinase A	5,5E-05	-2,1	G6PD	Glucose-6-phosphate dehydrogenase	4,2E-04	-2,3
FOXM1	Forkhead box M1	6,9E-05	-2,2	IGK@	Immunoglobulin kappa locus	4,3E-04	-2,8
LOC652493	LOC652493	1,1E-04	-2,9	IGHA1	Immunoglobulin heavy constant alpha 1	5,2E-04	-2,5
NUF2	NDC80 kinetochore complex component, homolog	1,2E-04	-2,1	KIF18A	Kinesin family member 18A	5,2E-04	-2,2
DTL	Denticleless homolog	1,2E-04	-2,1	IGKC	Immunoglobulin kappa constant	5,3E-04	-2,7
PLS1	Plastin 1 (I isoform)	1,3E-04	-2,0	CCNB2	Cyclin B2	5,5E-04	-2,5
KIF20A	Kinesin family member 20A	1,3E-04	-2,3	AKR1C3	Aldo-keto reductase family 1, member C3	5,5E-04	-2,1
MELK	Maternal embryonic leucine zipper kinase	1,5E-04	-2,2	NCAPG	Non-SMC condensin I complex, subunit G	5,9E-04	-2,3
IGLJ3	Immunoglobulin lambda joining 3	1,8E-04	-2,9	CENPF	Centromere protein F	7,7E-04	-2,3
LRIG3	Leucine-rich repeats and immunoglobulin-like domains 3	1,8E-04	-2,2	TPX2	TPX2, microtubule-associated, homolog	8,3E-04	-2,4
ASPM	Asp (abnormal spindle) homolog	2,0E-04	-2,5	CCDC102B	Coiled-coil domain containing 102B	8,8E-04	-2,2
BUB1	Budding uninhibited by benzimidazoles 1 homolog	2,1E-04	-2,3	CYP24A1	Cytochrome P450, family 24, subfamily A, polypeptide 1	9,0E-04	-2,3
SPAG5	Sperm associated antigen 5	2,1E-04	-2,1	LUM	Lumican	9,5E-04	-3,4
CCNB1	Cyclin B1	2,2E-04	-2,5	HELLS	Helicase, lymphoid-specific	9,7E-04	-2,0
IGHM	Immunoglobulin heavy constant mu	2,4E-04	-3,1	CDC2	Cell division cycle 2	9,8E-04	-2,4
GSDMC	Gasdermin C	2,6E-04	-2,0	LOC100290146	LOC100290146	1,0E-03	-2,4
BUB1B	Budding uninhibited by benzimidazoles 1 homolog	2,6E-04	-2,2	KIAA0101	KIAA0101	1,1E-03	-2,5
CEACAM5	Carcinoembryonic antigen-related cell adhesion molecule 5	2,7E-04	-3,8	IGKV3D-11	Immunoglobulin kappa variable 3D-11	1,1E-03	-3,0
ARHGAP11A	Rho GTPase activating protein 11A	2,7E-04	-2,3	LOC642838	LOC642838	1,1E-03	-2,2

FC, fold-change

Continued: Supplementary Table 2. Genes down-regulated by hypoxia

Gene symbol	Gene name	*P*	FC	Gene symbol	Gene name	*P*	FC
VSIG6	V-set and immunoglobulin domain containing 6	1,1E-03	-2,9	KIAA1199	KIAA1199	1,7E-03	-2,8
IL7R	Interleukin 7 receptor	1,1E-03	-2,1	SFTPB	Surfactant protein B	1,8E-03	-2,9
KIF4A	Kinesin family member 4A	1,2E-03	-2,2	SULF1	Sulfatase 1	1,9E-03	-2,7
SLC27A2	Solute carrier family 27 (fatty acid transporter), member 27	1,3E-03	-2,0	CLDN10	Claudin 10	2,0E-03	-2,4
KIF23	Kinesin family member 23	1,3E-03	-2,2	TMPRSS4	Transmembrane protease, serine 4	2,2E-03	-2,1
FAM111B	Family with sequence similarity 111, member B	1,3E-03	-2,1	CHI3L1	Chitinase 3-like 1	2,4E-03	-2,1
SLC34A2	Solute carrier family 34 (sodium phosphate), member 2	1,3E-03	-2,8	HMMR	Hyaluronan-mediated motility receptor	2,7E-03	-2,0
CENPI	Centromere protein I	1,3E-03	-2,6	CKMT1A	Creatine kinase, mitochondrial 1A	2,7E-03	-2,1
FAM46C	Family with sequence similarity 46, member C	1,4E-03	-2,2	PI15	Peptidase inhibitor 15	3,4E-03	-3,4
MKI67	Antigen identified by monoclonal antibody Ki-67	1,4E-03	-2,2	LAMB1	Laminin, beta 1	3,7E-03	-2,1
NAPEPLD	N-acyl phosphatidylethanolamine phospholipase D	1,5E-03	-2,2	LOC100293539	LOC100293539	4,1E-03	-2,7
CENPE	Centromere protein E	1,6E-03	-2,1				

FC, fold-change
