# Supplementary material for: Hypoxia increases membrane metallo-endopeptidase expression in a novel lung cancer ex vivo model – role of tumor stroma cells
Source: BMC Cancer. 2014 Jan 25;14:40. doi: 10.1186/1471-2407-14-40 (PMC3905926; doi:10.1186/1471-2407-14-40)

Supplementary Figure
Leithner et al.

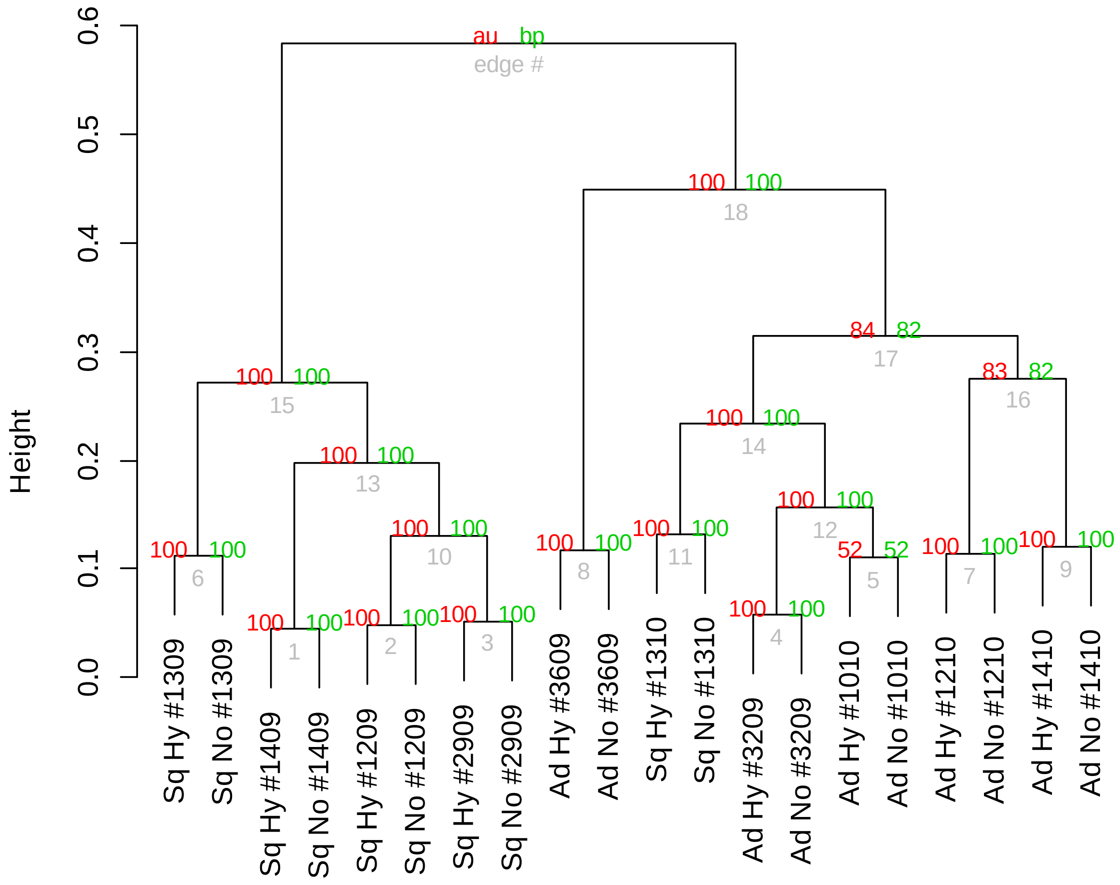

Supplement: Additional file 3: Figure S1 — Hierarchical clustering of gene expression profiles including all genes. P-values were calculated with pvclust based on 1000 bootstrap replications in 1426 selected genes with highest variability. In this software P-values greater than 95% are considered significant. Bootstrap P-values (bp) are shown in green. Asymptotically unbiased P-values (au) are shown in red. Cluster numbers (edge #) are shown in grey. The most similar pairs of arrays (shortest dendrogram branches) were the hypoxic and normoxic fragments from each patient. This close similarity was significant in 9 of 10 patients. Ad, adenocarcinoma; Sq, squamous cell carcinoma; No, normoxia; Hy, hypoxia. [file 1471-2407-14-40-S3.pdf]
